# Comparison of surgical and non-surgical orthodontic treatment approaches on occlusal and cephalometric outcomes in patients with Class II Division I malocclusions

**DOI:** 10.1186/s40510-017-0171-3

**Published:** 2017-07-03

**Authors:** Sheila Daniels, Patrick Brady, Arya Daniels, Stacey Howes, Kyungsup Shin, Satheesh Elangovan, Veerasathpurush Allareddy

**Affiliations:** 1 Department of Orthodontics, College of Dentistry and Dental Clinics, The University of Iowa, Iowa City, IA USA; 2 University of Michigan, Ann Arbor, MI USA; 3 College of Dentistry and Dental Clinics, The University of Iowa, Iowa City, IA USA; 4 Department of Periodontics, College of Dentistry and Dental Clinics, The University of Iowa, Iowa City, IA USA

## Abstract

**Background:**

This study aimed to examine end-of-treatment outcomes of severe Class II Division I malocclusion patients treated with surgical or non-surgical approaches. This study tests the hypotheses that occlusal outcomes (ABO-OGS) and cephalometric outcomes differ between these groups.

**Methods:**

A total of 60 patients were included: 20 of which underwent surgical correction and 40 of which did not. Cast grading of initial and final study models was performed and information was gathered from pre- to post-treatment cephalometric radiographs. The end-of-treatment ABO-OGS and cephalometric outcomes were compared to Mann-Whitney *U* tests and multivariable linear regression models.

**Results:**

Following adjustment for multiple confounders (age, gender, complexity of case, and skeletal patterns), the final deband score (ABO-OGS) was similar for both groups (23.8 for surgical group versus 22.5 for non-surgical group). Those treated surgically had a significantly larger reduction in ANB angle, 3.4° reduction versus 1.5° reduction in the non-surgical group (*p* = 0.002). The surgical group also showed increased maxillary incisor proclination (*p* = 0.001) compared to the non-surgical group. This might be attributed to retroclination of maxillary incisors during treatment selection in the non-surgical group—namely, extraction of premolars to mask the discrepancy.

**Conclusions:**

Those treated surgically had a significantly larger reduction in ANB angle and increased maxillary incisor proclination compared to those treated non-surgically with no significant changes in occlusal outcomes.

## Background

Class II Division I malocclusions typically manifest with increased overjet and retrognathic mandibles. Of Class II malocclusions, there are two subcategories: Division I (characterized by increased overjet and a retrognathic mandible) and Division II (in which maxillary lateral incisors or canines are proclined relative to the central incisors). Class II Division I malocclusions are the more common of the two in the European population [1]. National estimates in the USA indicate that 23% of children, 15% of youths, and 13% of adults have a discrepancy of 5 mm or more in overjet alone, thereby signifying a Class II Division I malocclusion [1]. Left untreated, Class II malocclusions can pose a variety of complications both present and future including those in the functional, psychological, and sociological realms [2, 3].

Treatment options for Class II Division 1 malocclusions are three-pronged: orthopedic growth modification, masking with extractions of premolars, and orthognathic surgery. Each option has been proven to be an effective means of treatment [4–17]. The decision as to which path to take depends on a variety of factors: time (as in, age of patient) and magnitude (amount of discrepancy: mild, moderate, or severe) [18]. A significant skeletal component is usually present in severe Class II Division 1 malocclusions. In these cases, the ideal method of treatment is orthodontic treatment in conjunction with orthognathic surgery as this is the only treatment which addresses the skeletal base discrepancy. However, due to finances or personal preference, patients are not always accepting of this option. In these situations, one of the other modes of treatment may be attempted in lieu of orthognathic surgery. In a younger patient, many orthopedic options achieve good facial harmony. However, while a masking treatment can address occlusal discrepancies, it will not improve skeletal position and therefore profile esthetics [17, 19–26].

Depending on the type of Class II corrector used (for example: Herbst, Twin Block, Headgear, or Forsus appliances), the end of treatment skeletal outcomes could vary [27–31]. Despite the fact that, especially in the USA, Class II malocclusions are possibly the most common malocclusions encountered by practitioners in private practice and in residency programs, there is little agreement on the best practice modality. This could be because treatment is multi-factorial, depending on age, timing of treatment, and patient concerns and desires. There is a paucity of studies that have compared outcomes of surgical versus non-surgical treatment of adolescent patients [32]. This is an important age to assess treatment outcomes because it is one of the most common ages for orthodontic treatment and treatment options might be confined depending on completion of the pubertal growth spurt.

The objective of the present study is to examine end-of-treatment cast-based and cepaholometric outcomes in patients with Class II Division I malocclusions treated orthodontically in conjunction with orthognathic surgery or without any orthognathic surgery. The study tests the hypothesis that end-of-treatment outcomes differ between the two treatment approaches.

## Methods

### Study design and participants

Inclusion criteria were as follows:

1. Class II Division 1 malocclusion (Class II molar relationship with proclined upper incisors)
2. Class II molar relationship
3. Initial overjet of ≥6 mm when measured on casts
4. Patient was debanded between ages 13 and <20 years of age
5. Treatment types (2): non-surgical orthodontic-only or a combination of orthodontics/surgical treatment
6. Availability of full records

Patients with craniofacial anomalies or syndromes were not included in the study.

### Sample size estimation

The sample size estimation for this study was based on the cephalometric measurements presented by Proffit et al [22] and the American Board of Orthodontics-Cast Occlusal Grading System (ABO-COGS) scores presented in the report by Cansunar and Uysal [33]. We deemed a 1- to 2-point difference in cephalometric outcomes between the surgical and non-surgical groups to be clinically significant. The mean ABO-COGS score in the study by Cansunar and Uysal ranged from 16.80 (standard deviation of 8.54) to 19.05 (standard deviation of 8.41) [33]. We deemed a one-standard deviation in ABO-COGS (between surgical and non-surgical groups) to be clinically significant. We set the alpha at 0.007 (to account for multiple testings) and power at 80%. Two-sided tests were to be used. Based on our sample size and power calculations, we estimated that each group should have 18 (for ABO-COGS scores) to 20 patients (for cephalometric variables). We planned on including 20 patients in the surgical group and 40 patients in the non-surgical group. We intentionally doubled the number of patients in the non-surgical group as this group is likely to have a wider range of biomechanical strategies (extraction, non-extraction, use of functional appliances, etc.) and a larger sample size would enable us to examine within group variations in outcomes.

### Key study variables

**Fig. 1:**
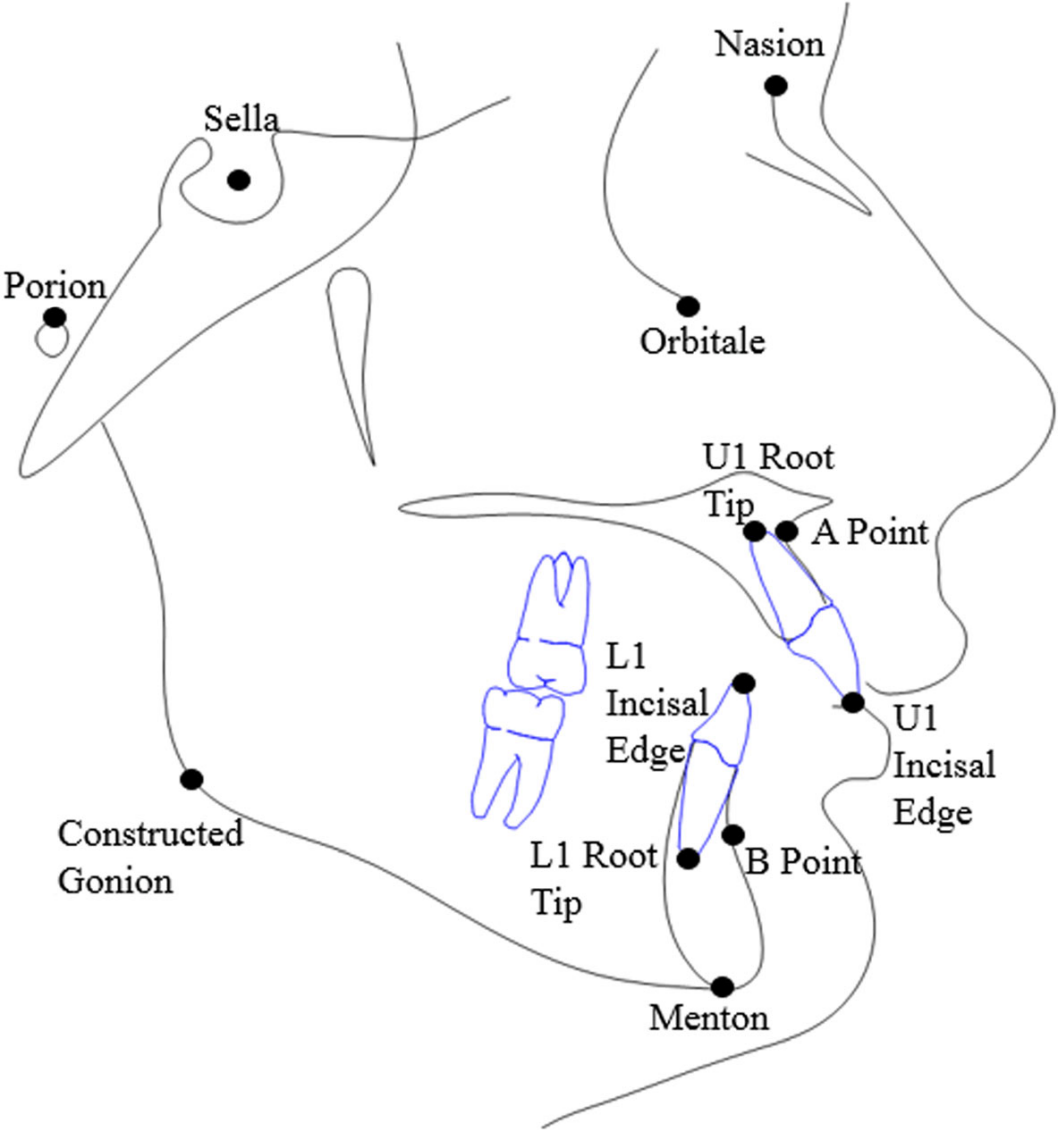
Cephalometric landmarks

**Fig. 2:**
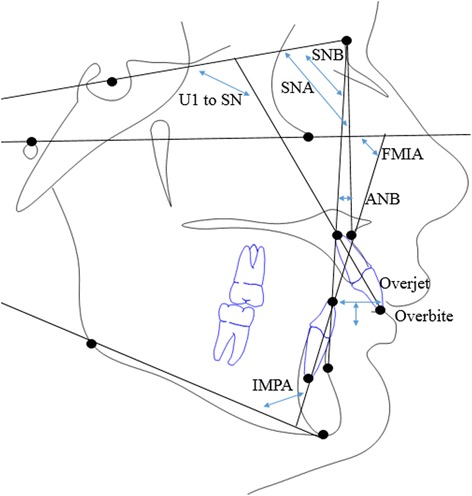
Angular and linear cephalometric measurements

Treatment data was gathered from all subjects. Initial and final lateral cephalometric radiographs were scanned into Dolphin Imaging software. The following cephalometric landmarks were traced and used for recording measurements: Sella, Porion, Orbitale, Nasion, A Point, B Point, U1 Incisal Edge, U1 Root Tip, L1 Incisal Edge, L1 Root Tip, Menton, and Constructed Gonion. Figures 1 and 2 provide a visual representation of these landmarks.

Cast grading was performed on pre- and post-treatment casts. Initial casts were graded using parameters determined by the ABO Initial Discrepancy Index Form (DI) (which is used to quantify the difficulty of an untreated case). Final casts were graded using the Final Cast Grading Form, also provided by the ABO, which provides a numerical representation of the finish of cases—higher numbers indicated more occlusal discrepancies in a finished case.

### Outcomes examined

Outcomes gathered in this study were as follows: deband lateral cephalometric outcomes (ANB, FMIA, IMPA, U1 to SN, overbite, overjet), cast occlusion grading outcomes (measured through the ABO-COGS), and retention protocol. Independent variables in this study were as follows: the type of treatment (surgical versus non-surgical), the initial discrepancy index (DI), initial cephalometric variables (ANB, FMIA, IMPA, U1 to SN, overbite, overjet), starting age of treatment, and gender.

### Examiner reliability

Inter-examiner and intra-examiner reliability analyses were performed using intra-class correlation coefficients (Cronbach alpha values) for each of the outcome variables. To compute intra-examiner reliability, one researcher measured initial casts and final casts for 20 cases two times within a 1-week interval to over 0.90 positive correlation. Inter-examiner reliability was performed between two different examiners. Both examiners used the initial discrepancy index form provided by the ABO and also the ABO Cast Grading form, which details instructions for cast grading at deband. In addition, both examiners took the same online tutorial for final cast grading, thereby having the same degree of training prior to measuring data. Both examiners were blinded with regard to the cases whether they were treated surgically or non-surgically. Correlation for inter-examiner reliability was >0.90. Cephalometric tracing also was reported with >0.90 correlation found for both intra- and inter-examiner reliability: two examiners independently traced the same ten radiographs two times over the course of two consecutive weeks (intra-examiner) and a second examiner traced the same ten later to compare results (inter-examiner).

### Statistical analysis

The baseline descriptives and outcomes were compared between the two groups using Mann-Whitney tests. Multivariable linear regression analyses were performed to examine the association between treatment (surgical versus non-surgical orthodontic treatment) and final lateral cephalometric numbers (adjusted for initial cephalometric numbers, age at start of treatment, initial DI, gender) and ABO-COGS. The multivariable linear regression models were fit using the ordinary least squares method. Sensitivity analyses were conducted using propensity scoring approach to account for the non-randomized nature of treatment assignment (surgical versus non-surgical). In this approach, we first computed the probability of a patient having undergone surgical or non-surgical treatment approach by using patient level covariates (age at start of treatment, gender, initial discrepancy index, initial ANB angle, initial FMIA angle, initial IMPA angle, initial U1 to SN angle, initial overbite, and initial overjet) as predictors in a logistic regression model fit by the maximum likelihood method. This model fitness was assessed by the Hosmer and Lemeshow goodness-of-fit test. After confirming that the model fit was good (Hosmer and Lemeshow goodness-of-fit chi-square value was 3.10 and *p* = 0.93), we used the predicted probability (propensity score) of being treated surgically or non-surgically in the second stage model as a covariate. The second stage model was fit using generalized linear model (GLM) methods. In this model, the primary independent variable was the type of treatment (surgical or non-surgical) and the propensity score was used as a covariate along with all other patient level variables. This approach was used to account for imbalances in treatment groups and reduces bias by mimicking randomization of subjects into treatment groups (surgical or non-surgical) [34]. The end-of-treatment outcomes between the surgical and non-surgical groups were assessed by propensity score regression adjustment and propensity score stratification approaches. In the stratification approach, five bins (quintiles) were used to stratify the propensity scores and the quintile was used as a covariate in the regression models. All the regression models were assessed for their fitness’. Several sensitivity analyses with different mix of covariates were conducted and the best fitting models with the highest *R*-square values were presented in this study. Since seven different end-of-treatment outcomes were assessed, to account for multiple outcomes assessment and minimize type 1 errors, we set the *p* value to be deemed statistically significant at *p* < 0.007. For comparing the baseline descriptives between the surgical and non-surgical groups, a *p* value of <0.05 was deemed to be statistically significant. All statistical tests were two-sided. All statistical analyses were conducted by the SPSS version 23.0 (IBM Corp, New York City, NY) software.

## Results

After the records were gathered, 60 patients were identified which fulfilled the inclusion criteria: 40 non-surgical and 20 surgical cases were included in the study. The study cohort was comprised of 28 female patients (21 in the non-surgical group and 7 in the surgical group) and 32 male patients (19 in the non-surgical group and 13 in the surgical group). Two patients were identified as Hispanic and two as multi-racial (Caucasian-African American). The remaining 56 patients were Caucasian. The mean age of the surgical group at the start of treatment was 14.8 years (compared to 12.9 years in the non-surgical group) [*p* < 0.001]. The mean age of the surgical group at the end of treatment was 17.4 years (compared to 15.4 years in the non-surgical group) [*p* < 0.001]. The duration of treatment for the surgical group was 2.6 years (compared to 2.5 years in the non-surgical group).

The mean initial discrepancy index score in the surgical group was 28.1 (compared to 20 in the non-surgical group) [*p* = 0.008]. The final ABO-COGS deband score was 23.8 in the surgical group (compared to 22.5 in the non-surgical). Initial descriptives are summarized in Table 1.

**Table 1:** Comparison of descriptives between treatment groups

Characteristic	Non-surgical patients			Surgical patients			
	Mean	Median	Std. deviation	Mean	Median	Std. deviation	*p* value
Initial discrepancy index	20.0	18.5	6.8	28.1	25.0	13.8	0.008
Final ABO-COGS deband score	22.5	21.0	8.2	23.8	23.0	9.7	0.666
Initial crowding/spacing upper	0.1	0.0	3.1	−1.0	−0.7	6.0	0.415
Initial crowding/spacing lower	0.1	0.5	4.3	−3.4	−3.7	4.0	0.415
Starting age (months)	154.6	151.5	20.9	177.1	179.0	16.1	<0.0001
Starting age (years)	12.9	12.6	1.7	14.8	14.9	1.3	<0.0001
Deband age (months)	184.8	180.0	18.4	208.7	207.0	15.2	<0.0001
Deband age (years)	15.4	15.0	1.5	17.4	17.3	1.3	<0.0001
Treatment duration (years)	2.5	2.3	0.8	2.6	2.6	0.8	0.227
Treatment duration (months)	29.5	28.0	10.0	31.5	31.8	9.5	0.227
Initial SNA	78.6	78.4	3.6	78.3	78.1	2.6	0.820
Initial SNB	74.8	74.3	3.3	72.3	72.5	3.3	0.024
Initial ANB	3.9	4.1	1.8	6.0	6.0	2.1	0.001
Initial FMIA	60.4	60.0	7.2	60.8	59.4	9.3	0.969
Initial IMPA	95.5	94.4	6.6	91.7	90.5	8.4	0.068
Initial U1 to SN	107.6	107.7	6.4	105.7	107.1	9.2	0.666
Initial Ceph overbite (mm)	4.6	5.0	1.8	4.6	5.1	3.7	0.931
Initial Ceph overjet (mm)	8.1	8.3	2.0	10.1	9.4	2.6	0.007
Deband SNA	77.8	77.3	4.0	77.7	78.5	2.6	0.772
Deband SNB	75.3	74.9	4.2	75.1	75.6	3.6	0.944
Deband ANB	2.4	2.9	1.9	2.6	2.8	2.9	0.701
Deband FMIA	56.0	56.3	7.0	58.6	57.2	5.2	0.121
Deband IMPA	100.4	100.1	5.2	92.3	92.4	7.8	<0.0001
Deband U1 to SN	101.6	100.1	7.6	102.7	101.9	10.2	0.772
Deband Ceph overbite (mm)	1.8	2.0	0.7	1.5	1.7	1.0	0.146
Deband Ceph overjet (mm)	2.9	2.8	1.2	3.1	2.8	0.9	0.354
Casts initial overjet (mm)	8.3	8.3	1.5	10.1	10.0	2.3	0.002
Cast initial overbite (mm)	4.6	5.0	1.8	3.9	4.5	2.8	0.080

Final treatment plans in the non-surgical group is shown in Table 2. Of the 20 surgical candidates, surgical breakdown was as follows: one piece maxillary impaction (2), BSSO mandibular advancement only (*n* = 16), and bi-maxillary surgery (*n* = 2). Comprehensive final treatment plans in the surgical group is depicted in Table 3.

**Table 2:** Final treatment plan in the non-surgical treatment group

Overall treatment type	Number of patients
Headgear only	2
Headgear and elastic wear	14
Headgear and upper first bicuspid extractions	3
One upper biscupid only	1
Upper first bicuspid extractions only	3
Four bicuspid extractions only	1
Headgear in addition to upper premolar extractions and elastic wear	2
Headgear and forsus	3
Forsus correction only	1
Herbst and elastic wear	3
Herbst followed by headgear and elastic wear to hold correction	1
Headgear as anchorage in conjunction with two bicuspid extractions	1
Headgear as anchorage in conjunction with four bicuspid extractions	1
Extraction of upper first premolars with TADs	1
Started on HG and declines surgery	1
Deband once alignment was achieved	1
HG then elastics off TADs	1

**Table 3:** Final treatment plan in the surgical treatment group

Overall treatment type	Number of patients
Extraction of four premolars with HG for anchorage followed by a surgery	4
Non-extraction BSSO advancement and genioplasty	2
Maxillary impaction and BSSO advancement	1
RME in conjunction with extraction of upper premolars and a BSSO/genioplasty	1
RME with 4 premolar extractions with BSSO/genioplasty	1
RME non-extraction with a BSSO/genioplasty	1
RME with four premolar extractions with maxillary impaction	1
Extraction of all second premolars with a BSSO advancement	1
RME with extraction of lower first premolars then a BSSO	1
RME with extraction of lower first premolars then a BSSO with genioplasty	1
SARME with extraction of lower first premolars followed by a BSSO/genioplasty	1
Extraction of four premolars with HG for anchorage followed by a surgery	1
Extraction of lower first premolars and BSSO only	1
Unspecified surgery	3

Both groups utilized TADs or HG for anchorage purposes. The breakdown in each group was as follows: non-surgical group—headgear (*n* = 29), headgear and TADs (1), TADs with no headgear (*n* = 3), and neither headgear nor TADs (*n* = 7)—and surgical group—headgear (*n* = 3), TADs in the lower arch only (*n* = 1), neither headgear nor TADs (*n* = 16).

In the non-surgical group, the retention options delivered were as follows: fixed (bonded) retainers on the lingual aspect of maxillary central incisors along with Hawley retainers (*n* = 2), Hawley retainers only (2), Hawley retainer and bonded lower retainer (2), Hawley retainers only (33), and tooth positioner followed by Hawley retainers (1). In the surgical group, the retention protocols included the following: Hawley retainers only (17), tooth positioner and Hawley retainers (2), and one patient was given a tooth positioner and never returned for the Hawley retainer.

Consent deband, indicating premature treatment completion, was tracked in each group. Of the non-surgical patients, 32 did not have a consent deband. The remaining 8 patients opted for consent deband: 1 finished with a crossbite, 1 consent debanded due to patient burnout, 1 decided to stop treatment as correction could not be achieved and would consider surgery or extractions at a later time point, and 5 consent debanded with no reason indicated. In the surgical group, 15 did not consent deband and the remaining 5 did. Consent deband with no reason indicated was done in 4 patients and 1 with the reason being that they did not want to wear their elastics anymore.

Estimates from the multivariable linear regression models that were fit using the ordinary least squares methods are summarized in Table 4. After adjustment for all available patient level covariates (age at start of treatment, gender, initial discrepancy index, initial ANB angle, initial FMIA angle, initial IMPA angle, initial U1 to SN angle, initial overbite, and initial overjet), the ABO-COGS deband score in the surgical treatment group was 0.854 points lower than that in the non-surgical group. The deband ANB angle in the surgical treatment group was 2.24° lower than that in the non-surgical group and this was statistically significant (*p* = 0.002). The deband FMIA angle in the surgical treatment group was 0.649° more than that in the non-surgical group. The deband IMPA angle in the surgical treatment group was 3.32° lower than that in the non-surgical group. The deband upper incisor to SN plane angle in the surgical treatment group was 10.564° more than that in the non-surgical group and this was statistically significant (*p* = 0.001). The deband cephalometric overbite in the surgical treatment group was 0.606 mm lower than that in the non-surgical group. Deband cephalometric overjet in the surgical treatment group was 0.188 mm more than that in the non-surgical group. The results of the sensitivity analyses conducted using the propensity scoring techniques (propensity scoring regression and stratification models) are summarized in Table 4. Overall, the two propensity scoring techniques showed that the parameter estimates were consistent with those obtained by fitting with ordinary least squares approach. After adjustment for the propensity scores and patient level covariates, those treated surgically had significantly lower deband ANB angle and higher upper incisor to SN plane angle compared to those treated non-surgically.

**Table 4:** Estimates of lateral cephalometric outcomes from multivariable regression models

Primary independent variable	Outcomes	Multivariable regression models					
		Linear regression model fit with ordinary least squares regression approach^a^		Propensity score regression model fit with GLM method^b^		Propensity score stratification model fit with GLM method^c^	
		Parameter estimate	*p* value	Parameter estimate	*p* value	Parameter estimate	*p* value
Surgical treatment Versus non-surgical treatment (reference variable)	ABO-COGS deband score	−0.854	0.80	−0.562	0.89	−1.06	0.76
	Deband ANB angle	−2.24	0.002	−2.11	0.01	−2.40	0.001
	Deband FMIA angle	0.649	0.75	−0.35	0.89	0.765	0.72
	Deband IMPA angle	−3.321	0.09	−3.23	0.17	−3.50	0.08
	Deband upper incisor to SN plane angle	10.564	0.001	10.03	0.01	11.53	<0.001
	Deband overbite	−0.606	0.07	−0.570	0.16	−0.610	0.08
	Deband overjet	0.188	0.71	0.283	0.65	0.161	0.76

^a^In this model, the confounding effects of covariates (age at start of treatment, gender, initial discrepancy index, initial ANB angle, initial FMIA angle, initial IMPA angle, initial U1 to SN angle, initial overbite, and initial overjet) were adjusted. The linear regression models were fit using ordinary least squares regression approach

^b^A two-staged regression approach was used. In the first stage, propensity scores (predicted probability of a patient having orthognathic surgery) were computed by using covariates (age at start of treatment, gender, initial discrepancy index, initial ANB angle, initial FMIA angle, initial IMPA angle, initial U1 to SN angle, initial overbite, and initial overjet). In the second stage, the effect of surgical versus non-surgical treatment on outcomes was examined by GLM model in which the propensity score was used as continuous variable and was adjusted as a covariate along with all other covariates

^c^A two-staged regression approach was used. In the first stage, propensity scores (predicted probability of a patient having orthognathic surgery) were computed by using covariates (age at start of treatment, gender, initial discrepancy index, initial ANB angle, initial FMIA angle, initial IMPA angle, initial U1 to SN angle, initial overbite, and initial overjet). In the second stage, the effect of surgical versus non-surgical treatment on outcomes was examined by GLM model in which the propensity score was stratified into five bins (based on distribution of scores) and was adjusted as a covariate along with all other covariates

## Discussion

The present study is a retrospective analysis of consecutively treated patients with Class II Division I malocclusions. The goal of our study was to compare the end-of-treatment outcomes in patients with Class II Division I malocclusions who were treated surgically or non-surgically. Our findings showed that the cast-grading outcomes were similar between the surgical and the non-surgical treatment groups and certain end-of-treatment cephalometric values differed between the two groups. Our results showed that after adjustment for all available patient level covariates, the final mean ABO-COGS deband score in the surgical group was 0.854 points lower than that in the non-surgical group. The deband ANB angle in the surgical group was 2.24° lower than that in the non-surgical group. This indicates that the maxilla/mandible relationship improved in the surgical group to a greater extent. This can be expected since skeletal positions change with a surgical treatment. Both FMIA and IMPA angle give information about lower incisor position. The deband FMIA angle and the IMPA angle indicate the position of the mandibular incisors. Our study results showed that the FMIA angle was not significantly different between the two treatment groups. The deband IMPA was significantly higher for the non-surgical group (100.4°) compared to the surgical group (92.3°). However, this difference became statistically non-significant once age, gender initial discrepancy index, and other cephalometric variables were adjusted in the regression models. Following adjusting for all confounders, those treated surgically had 3.3° lower IMPA compared to those treated non-surgically. This indicates that the mandibular incisors were more upright at the end of treatment in the surgical group. We would expect these values to change according to which treatment plan, surgical or non-surgical, was chosen. For instance, if lower premolars were extracted before a mandibular advancement, we would expect some uprighting of the lower incisors during space closure. Also, initial crowding would have an effect on incisor position. If no extractions were done, the way to gain arch length to resolve lower anterior crowding is to procline the lower incisors. The deband upper incisors to SN plane angle, a measurement of upper incisor position, was shown to be 10.56° higher in the surgical treatment group (*p* = 0.001) after adjustment of all covariates in the regression model. We expected this finding because, if a surgical option cannot be entertained, a masking approach by extracting upper premolars is most likely considered instead. During space closure, there will be uprighting of the upper incisors, thereby leading to a decreased upper incisor to SN plane angle. This finding could also be explained in that for a surgical treatment option where teeth might not need to be extracted, if there is an existing upper anterior crowding, incisors will be proclined to gain space for alignment. Deband cephalometric overbite was found to be 0.606 mm lower in the surgical group while deband cephalometric overjet was shown to be 0.188 mm higher in the non-surgical treatment group after adjustment for all covariates in the regression models and these were not statistically significant.

One of the first pieces of literature analyzing need for orthognathic surgery based on severity was put forth by Proffit et al. in 1992 [22]. When reviewing an adolescent population treated non-surgically (through camouflage treatment) or surgically, Proffit et al. identified certain parameters which might be useful when deciding treatment. They evaluated cephalometric and cast measurement before and after treatment to determine efficacy of treatment. Our study found end-of-treatment occlusion to be similar in both groups. This was supported by work of Proffit et al. as well. Our study showed that overjet was slightly higher in the surgical group, which was not seen in the study by Proffit et al. This could be attributed to differing practitioners’ approach to treatment or variability in the success of the surgical treatment in respective surgical populations.

Mihalik et al. performed a long-term follow-up of Class II adults treated with camouflage treatment or surgical treatment and analyzed post-deband results [19]. Patients in this population were recalled 12 years after treatment. This group found that both groups showed acceptable correction of the malocclusion. This was echoed by our study which found ABO cast grading outcomes at the end of treatment was not significantly different between the surgical and non-surgical groups. At recall, Mahalik et al. reported that in both populations, overbite increased to a small extent and overjet increased in the surgical group by 10–20% [19]. Our study found that deband cephalometric overbite was lower in the surgical group (compared to non-surgical) and overjet was increased compared to the non-surgical group, although neither value was statistically significant. This might be expected because with camouflage treatment, as the upper incisors are retracted, overbite increases.

In an adolescent population (less than 20 years of age), Tulloch et al. discussed the difficulty in treatment planning as these patients might still be undergoing growth. This study emphasized that in severe cases, surgical treatment is most likely the best option [35]. They examined 500 patients in a study with similar inclusion criteria as ours. Patients were treated non-surgically or surgically and end-of-treatment outcomes were reviewed based on division into three categories: orthodontic success, orthodontic failure, and surgical success. This study assessed success of treatment through reduction in overjet to less than 4 mm [35]. Cephalometric radiographs were reviewed and patients were placed into two sub-groups based on gender. Initial ANB in this study was about 6°, similar to that in our study, but the initial overjet measurement is that both groups were significantly more (7.8 and 8.6 mm) when compared to our population (2.9 versus 3.1 mm). This study found that 98% of patients in their entire population did not meet their criteria for correction of overjet [35]. Since our study evaluated ANB change as a measure of AP correction, we were able to focus exclusively on skeletal position instead of tooth position. Tulloch concluded that neither gender nor age were associated with success of correction of overjet and concluded that more factors go into a “successful” or “unsuccessful” case than practitioners might think. Since these factors were held constant in our linear regression models, we were able to analyze differing variables without the risk of bias.

Kinzinger et al. studied outcomes in patients with Class II Division I malocclusions where in 60 young adults were evaluated after a surgical or non-surgical treatment [20]. Their results showed changes in all skeletal categories, as can be presumed because with this method of treatment, the skeletal base is being influenced directly. Each group in this study achieved a reduction in overjet. The surgical group was found to have significant protrusion of upper incisors, as did our research. This might be attributed to the biomechanical differences in treating a surgical case versus a non-surgical case. One might imagine that not only will a camouflage treatment increase overbite as incisors are retracted, but they will also upright. If a surgical patient has minimal crowding, it might not be outside the realm of possibility that the practitioner might choose to procline the upper incisors to allow for the alignment of the teeth before the patient is sent for surgery.

The findings of our study should be interpreted keeping the inherent limitations of retrospective studies in perspective. What we found is an association and not a true causal effect. Our analysis was limited to the variables we could gather from the treatment chart notes. The population in our study was relatively homogenous considering the location of the dental school and the population it serves. Consequently, our study results cannot be generalized to all Class II Division I malocclusions.

## Conclusions

We can conclude that amongst Class II Division I cases identified in this study, there were some differences in deband outcomes between non-surgical and surgical populations. Those treated surgically had a significantly larger reduction in ANB angle and increased maxillary incisor proclination compared to those treated non-surgically. Further information should be gathered at other institutions to compile a more diverse picture of successful treatment options in the Class II Division I population.
